# Rove beetle subtribes Quediina, Amblyopinina and Tanygnathinina: systematic changes affecting Central European fauna (Coleoptera, Staphylinidae, Staphylinini)

**DOI:** 10.3897/zookeys.162.2361

**Published:** 2012-01-05

**Authors:** Alexey Solodovnikov

**Affiliations:** 1 Department of Entomology, Zoological Museum (Natural History Museum of Denmark), Universitetsparken 15, Copenhagen 2100 Denmark

**Keywords:** Staphylinini, Quediina, Amblyopinina, Tanygnathinina, *Heterothops*, *Quedius*, *Velleius*, phylogeny, new synonyms, lectotype designations, misidentifications, Central Europe

## Abstract

In preparation for the new edition of the identification keys of rove beetles of Central Europe (Volume 4 of the “Die Käfer Mitteleuropas”), the following systematic problems affecting the Central European fauna of the tribe Staphylinini are addressed: phylogeny-based, new concepts for the subtribes Quediina and Amblyopinina; status of the subtribe Tanygnathinina; systematic position of the genus *Astrapaeus*; status of *Quedionuchus*, the subgenus of *Quedius*; identity of some species of *Quedius* and *Heterothops*. As a result, new wordwide and Central Europe-based diagnoses are given for the subtribes Quediina and Amblyopinina; earlier recognized but not widely accepted synonymies of the genera *Quedius* and *Velleius*, and of the species *Heterothops praevius* and *Heterothops niger*, are justified; new synonyms are established for: *Quedius pseudonigriceps* Reitter, 1909 (= *Quedius* *noricus* Bernhauer, 1927, **syn. n.**); *Quedius maurorufus* (Gravenhorst, 1806) (= *Quedius richteri* Korge, 1966, **syn. n.**); *Quedius suturalis* Kiesenwetter, 1845 (= *Quedius merlini* Drugmand & Bruge 1991, **syn. n.**); lectotypes are designated for *Quedius meridiocarpathicus* Smetana, 1958, *Quedius noricus* Bernhauer, 1927, and *Quedius pseudonigriceps* Reitter, 1909. As a result of synonymy of *Quedius* and *Velleius*, the following new combinations are proposed: *Quedius amamiensis* (Watanabe, 1990), **comb. n.**; *Quedius circumipectus* (Cho, 1996), **comb. n.**; *Quedius elongatus* (Naomi, 1986), **comb. n.**; *Quedius japonicus* (Watanabe, 1990), **comb. n.**; *Quedius pectinatus* (Sharp, 1874), **comb. n.**; *Quedius setosus* (Sharp, 1889), **comb. n.**; *Quedius simillimus* (Fairmaire, 1891), **comb. n.** As a result of new combinations, *Quedius japonicus* (Watanabe, 1990) (non *Quedius japonicus* Sharp, 1874) is replaced with the new name *Quedius watanabei* Solodovnikov, **nom. n.,** while *Quedius pectinatus* Lea, 1908 (non *Quedius pectinatus* (Sharp, 1874)) is replaced with the new name *Quedius arthuri* Solodovnikov, **nom. n.**

## Introduction

Central Europe (territories of Denmark, Germany, Poland, Benelux-states, Austria, Czech Republic, Slovakia and Switzerland) is a conventional area that has no integrity in terms of biogeography. But since this region has a strong common entomological tradition, the insect fauna of Central Europe is often viewed as such despite not being cohesive either zoogeographically or phylogenetically. Currently it is perhaps the best known entomofauna in the world as far as any other territory of comparable size is concerned. At least this is true for the beetle family Staphylinidae covered in the milestone volumes 4 and 5 of the well-known series “Die Käfer Mitteleuropas”. In the course of time however, the inevitable obsolescence of these reference books necessitates new editions. Gladly, a new version of the Volume 4 (Lohse 1964) was recently accomplished by an international team of authors led by German colleagues Volker Assing (Hannover) and Michael Schülke (Berlin) (Assing and Schülke 2012).

Being involved in that project as an author of the sections equivalent to “Quediini” and “Atanygnathinini” (Staphylininae) in Lohse (1964), I came across a necessity of publishing some formal taxonomic changes for the Central European fauna to be used in Assing and Schülke (2012). Also, some earlier published world-wide systematic work on Staphylinini (Solodovnikov and Newton 2005; Solodovnikov 2005, 2006; Solodovnikov and Schomann 2009; Chatzimanolis et al. 2010) that affected the Central European fauna, needed a concise digest specifically targeting a European user. All these issues are addressed in the present paper, and grouped in the following three categories: subtribal classification of Staphylinini; *Quedius*-complex; and species-level problems in *Heterothops* and *Quedius*.

## Material and methods

Material examined in this paper came from the following institutional and private collections:

FMNH Field Museum of Natural History, Chicago, U.S.A (M. Thayer, J. Boone)


HNHM Hungarian Museum of Natural History, Budapest, Hungary (G. Makranczy)


NHMW Vienna Museum of Natural History, Austria (H. Schillhammer)


NMPC National Museum, Prague, Czech Republic (Jiř˘ı Hájek)


ZMUC Zoological Museum of the University of Copenhagen (part of the Danish Natural History Museum), Denmark


cAS Private collection of A. Smetana (Ottawa)


cKrg Private collection of H. Korge (Berlin)


cSch Private collection of M. Schülke (Berlin)


## Subtribal classification of Staphylinini

With more than 200 genera and more than 5,000 species worldwide, Staphylinini is one of the largest tribes of rove beetles. As mentioned in recent works (e.g., Smetana and Davies 2000; Solodovnikov 2006; Solodovnikov and Schomann 2009; Chatzimanolis et al. 2010), the supra-generic classification of Staphylinini currently in use (e.g., Herman 2001; Newton and Thayer 2005; Bouchard et al. 2011) needs modification. Although many aspects of the phylogeny of Staphylinini are still unclear, certain parts of it are already resolved and translated into a classification. Some aspects affecting the fauna of Central Europe are summarized here.

### On the systematic position of the genus *Astrapaeus* Gravenhorst, 1802

Both morphology- and molecular-based analyses, no matter how they disagree in detail, place certain members of the conventional subtribe “Quediina” (genera *Afroquedius* Solodovnikov, 2006*, Astrapeus* Gravenhorst, 1802*, Parisanopus* Brèthes, 1900 and *Valdiviodes* Smetana, 1981) as basal lineages of Staphylinini (see for example fig. 6 in Solodovnikov 2006, fig. 1 in Solodovnikov and Schomann 2009; and fig. 1 in Chatzimanolis et al. 2010). These genera are species-poor and have narrow distributions scattered around the globe, such evidence also suggesting their ancient, relict nature among Staphylinini. Contrary to the formal classification where they are placed in the subtribe Quediina, neither of them form a monophyletic group with the “core” clade of “*Quediina”* (the monophyletic part of the conventional Quediina hosting the type species of *Quedius*; e.g, the clade marked in red in fig. 1 in Solodovnikov and Schomann 2009). To balance the formal classification of Staphylinini with the underlying phylogeny, a series of monobasic supra-generic groups (possibly subtribes) has to be erected for those isolated basal genera. However, to avoid premature creation of several new family-group names when the phylogeny of the entire Staphylinini is not stabilized yet, Chatzimanolis et al. (2010, table 1) classified such genera as *incertae sedis* within Staphylinini. Since *Astrapeus* is the only genus in Central Europe that falls in this category, and the European authors are used to its placement in Quediina, the species *Astrapaeus* *ulmi* (Rossi, 1790) is still listed as a member of that subtribe in the new edition of the “Die Käfer Mitteleuropas” (Assing and Schülke 2012). Unlike the specialized phylogenetic paper of Chatzimanolis et al. (2010), the keys to Central European fauna is a practical tool, limited geographically, but targeting a very broad scope of users with varying taxonomic background. Therefore, for those keys, the exact and familiar classification of a phylogenetically unstable taxon seems a more useful solution, as opposed to its uncertain position, even though the latter may reflect the current phylogenetic knowledge more accurately.

### New limits and diagnosis of the subtribe Quediina Kraatz, 1857

Similarly to basal groups like *Astrapaeus*, some other genera across Staphylinini display “*Quedius*-like” habitus. That similarity is mostly caused by their deflexed hypomera of pronotum and variously shaped “infraorbital ridges” (the latter often combine non-homologous structures, as discussed in Solodovnikov 2006). The “*Quedius*-like” habitus of unrelated Staphylinini misled systematists who gradually inflated Quediina to a largely polyphyletic taxon. Contrary to the currently accepted classification but according to the abovementioned new phylogenetic data, the limits of the subtribe Quediina should be restricted to the north temperate Holarctic core of the current genus *Quedius*, plus some other, mostly Holarctic, smaller genera of the traditional “Quediina”. An example of Quediina in such new definition is marked by red in fig. 1 in Solodovnikov and Schomann (2009), while the entire list of genera of the newly defined subtribe Quediina is provided in the table 1 in Chatzimanolis et al. (2010). Within the Central European fauna all species of the genus *Quedius* (including *Velleius* as a synonym of *Quedius*, seebelow), as well as genera *Euryporus* and *Acylophorus*, belong to Quediina in the newly defined sense. But the genus *Heterothops* that also occurs in Central Europe and that traditionally stayed in the subtribe “Quediina”, however, belongs to the subtribe Amblyopinina, also in a newly defined sense (see below). To accommodate these changes, new global and regional diagnoses of Quediina are here provided.

*Quediina: new diagnosis based on world fauna*. Small to medium size beetles with pronotum having deflexed hypomera and thus not visible in lateral view; head with well-developed infraorbital ridges (as defined in Smetana and Davies 2000) extending from neck towards base of mandibles and often reaching the latter; tarsal formula 5-5-5; mesoscutellum with two basal carinae (in normal position that part of mesoscutellum is hidden under base of pronotum); aedeagus of variable shape, but with paramere never very closely attached or fused to median lobe, mostly with distinct, heavily sclerotized sensory peg setae.

Except a few (mostly montane) species extending into (sub)tropical latitudes of the Oriental and Neotropical regions, and some adventive species that occur nearly world-wide, the group is restricted to the north temperate zone of the Holarctic region and is markedly absent in the Sub-Saharan Africa. Many Quediina are confined to leaf litter of the north temperate forests, some also occur in ground-based debris of various open landscapes.

*Quediina: diagnosis based on Central European fauna*. Small to medium size beetles; head with well-developed infraorbital ridges; pronotum with deflexed hypomera and thus not visible in lateral view, on disc with 2–4 punctures in dorsal rows; tarsal formula 5-5-5; apical segment of maxillary and labial palps never very narrow or aciculate, mostly (but not always) fusiform with more or less truncate apex; aedeagus with well developed paramere that is separated from the median lobe along most of its length, mostly with sensory peg setae.

### New limits and diagnosis of the subtribe Amblyopinina Seevers, 1944

Along with the new definition of Quediina, the mentioned phylogenetic studies reveal an earlier unrecognized monophyletic lineage that consists of: some south temperate genera of Staphylinini most of which were in the conventional subtribe “Quediina” (for their list see table 1 in Chatzimanolis et al. 2010); many Staphylinini species from Australia and New Zealand currently misplaced in the genus *Quedius* (e.g., represented by *Quedius calogaster* Lea, 1929 in the analysis of Solodovnikov and Schomann 2009); and several genera of truly remarkable staphylinids from South America and Australia (members of the subtribe Amblyopinina Seevers 1944 in the conventional system, e.g., Herman 2001). As far as the Central European fauna is concerned, it is only the genus *Heterothops* (globally distributed, poorly defined genus, for details see Solodovnikov and Schomann 2009) that belongs to this lineage. Since Amblyopinina Seevers, 1944 is the oldest available family-group name for this newly found large monophyletic lineage, its meaning has to be expanded far beyond the initial scope that included only highly specialized “very exotic” Neotropical and Australian symbionts of small mammals. The strongly modified morphology of the latter is an adaptation to a very special habitat like the fur of a mammal body; such strong autapomorhy simply disguised sister relationships of these beetles for decades. Moreover, it is apparent that the symbiosis with mammals and associated specialized morphology may have originated independently in several lineages of free living “usual *Quedius*-like” south temperate Amblyopinina (Ashe and Timm 1988). Following the discussed phylogenetic results, and in agreement with the here provided new diagnosis of Amblyopinina in Assing and Schülke (2012), *Heterothops* is treated used in the subtribe Amblyopinina, not in Quediina.

*Amblyopinina: new diagnosis based on world fauna*. Small to medium size beetles with pronotum having deflexed hypomera and thus not visible in lateral view; tarsal formula 5-5-5; mesoscutellum with one basal carina (in normal position that part of mesoscutellum is hidden under base of pronotum); aedeagus: paramere longer than, and closely attached to, median lobe; often median lobe relatively poorly developed or, in the ultimate case of *Heterothops*, reduced and entirely fused to strongly developed paramere.

Except the global genus *Heterothops*, the group is restricted to the southern hemisphere, and is especially species-rich and abundant in leaf and log litter of the south temperate and subtropical forests of southern South America, Australia, New Zealand, and less so in Papua New Guinea and New Caledonia. A few genera of Amblyopinina, possibly not closely related to each other, are symbionts of mammals and have peculiar “ectoparasitic” morphology.

*Amblyopinina: diagnosis based on Central European fauna*. Small beetles with pronotum having deflexed hypomera not visible in lateral view, disc of pronotum with two punctures in dorsal row; tarsal formula 5-5-5; apical segment of maxillary and labial palps very narrow, aciculate, at base much narrower than their respective penultimate segments; aedeagus with median lobe reduced and entirely fused to strongly developed paramere.

### Status of the subtribe Tanygnathinina Reitter, 1909

In connection with the discussion about Amblyopinina in the new sense, the systematic position of the genus *Atanygnathus* Jakobson, 1909, represented in Central Europe by a single species *Atanygnathus terminalis* (Erichson, 1839), should be also commented. Adult and larval morphology of *Atanygnathus* is very peculiar (Solodovnikov 2005; Staniec 2005), but according to the morphology-based phylogenetic analyses (Solodovnikov 2006; Solodovnikov and Schomann 2009), these peculiarities apparently are autapomorphies, while the genus shares synapomorphies with the above discussed large south temperate lineage Amblyopinina. Contrary to morphology though, the molecular analysis (Chatzimanolis et al. 2010) did not support affiliation of *Atanygnathus* with that group and, at the same time, did not suggest a plausible alternative placement. Conflicts among various datasets, especially as different as animal morphology and DNA-sequences, are not unusual in systematic biology. Given a very high impact of morphology on practical systematics, and instability of molecular phylogenies when they are based on few genes (as opposed to generally more robust multigene phylogenies), a morphology-based solution for the systematic placement of a taxon would have been given a priority over a conflicting hypothesis that is based on limited molecular dataset. But, as far as *Atanygnathus* is concerned, there are two practical considerations against the placement of *Atanygnathus* in Amblyopinina. Firstly, immediate acceptance of the morphology-based hypothesis would necessitate the synonymy of the family-group names Tanygnathinina Reitter, 1909 and Amblyopinina Seevers, 1944, where the former name would be valid due to its priority while being tied to the phylogenetically most unstable taxon. Secondly, the monobasic Tanygnathininacan be easily characterized and keyed out by striking autapomorphiesof *Atanygnathus*: very elongate mouthparts and tarsal formula 5-4-4, both features unique among Staphylinini. Inclusion of *Atanygnathus* into Amblyopinina, on the contrary, would diffuse the diagnosis of the latter subtribe. As a result, a separate monobasic subtribe Tanygnathinina is currently maintained for that genus, also in Assing and Schülke (2012).

## *Quedius*–complex

One of the biggest systematic problems at the genus level within the tribe Staphylinini is the so-called “*Quedius*-complex” (Solodovnikov 2006). As it stands now (for example, Herman 2001; Newton and Thayer 2005), the genus *Quedius* is highly polyphyletic and lacks a consistent intrageneric division. Operational species groups in *Quedius* were defined only for some regional faunas like America North of Mexico, and parts of the Palearctic and Oriental regions, while the originally very inconsistent subgeneric division, although once improved by Smetana (1971) for the Holarctic fauna, still needs a rigorous phylogenetic and broader overview. As a result, there remains a plethora of genus-group taxa within and around *Quedius*, whose status remains controversial. With respect to Central European fauna, *Quedionuchus* Sharp, 1884 and *Velleius* Leach, 1819 are such groups.

### On the status of *Quedionuchus* Sharp, 1884

*Quedionuchus* was originally established as a genus (Sharp 1884) (with the type species *Quedius impunctus* Solsky, 1868, designated by Blackwelder 1952). Eventually various European authors downgraded *Quedionuchus* to a subgenus of *Quedius* and expanded its limits to include also some species of *Distichalius,* another subgenus of *Quedius* (Smetana 1971). Smetana (1971) corrected the volume of *Quedionuchus* by removing members of *Distichalius* from the former, but he left *Quedionuchus* as a subgenus of *Quedius*. Analysis in Solodovnikov (2006) placed *Quedionuchus* outside *Quedius*, suggesting that a separate generic status for the former would be a better solution. Because the formal reclassification of the “*Quedius*-complex”is pending a broader study, in Assing and Schülke (2012) the traditional subgeneric status of *Quedionuchus* is maintained for practical reasons.

### Synonymy of *Quedius* Stephens, 1829 and *Velleius* Leach, 1819

Leach (1819) described the genus *Velleius* to accommodate two species, *Staphylinus* *dilatatus* Fabricius, 1787 and *Staphylinus concolor* Marsham, 1802 (currently a synonym of *Velleius dilatatus* (F.)), the latter species subsequently (Westwood 1838) designated as a type species. Although all eight currently known species of *Velleius* (Herman 2001; Smetana 2004; new combinations below) share characteristic large size and pectinate antennae, doubts regarding a separate generic status for this group were expressed by a number of earlier authors who treated *Velleius* as a synonym of *Quedius* (e.g., Erichson 1839; Lacordaire 1854; Kraatz 1857; Schaum 1859). Also Smetana (1988) pointed out a case when it was difficult to assign a species, *Quedius inquietus* (Champion, 1925) (originally described as *Velleius*), to either *Velleius* or *Microsaurus*, a subgenus of *Quedius*. The habitus, taxonomically important chaetotaxy and aedeagus of *Velleius* are essentially the same as in *Microsaurus*. The larva of *Velleius* is *Quedius*-like (Paulian 1941; Strassen 1957; Pototskaya 1967; data matrix in Pietrykowska-Tudruj et al. 2011). Molecular-based phylogenetic analysis (Chatzimanolis et al. 2010) also placed species of *Velleius* nested within *Quedius* (*Microsaurus*). Even a peculiar biology, known for *Velleius dilatatus* (larvae of this species live in the nests of the European hornet *Vespa crabro* (e.g., Strassen 1957)) is just a strongly expressed case of an overall evolutionary trend towards nidicoly seen in many other species of *Microsaurus*. Therefore, following some earlier authors, *Velleius* and *Quedius* should be considered as synonyms, that is also followed in Assing and Schülke (2012). Because *Quedius* is a much more species-rich and abundant genus than *Velleius*, in the interests of stability of the zoological nomenclature, an application to the International Committee for the Zoological Nomenclature has been prepared to suppress the Priority Rule and give precedence to the younger generic name *Quedius* Stephens, 1829 over the older generic name *Velleius* Leach, 1819. Since the species *Velleius dilatatus* (F.) was used in the combination with the genus *Quedius* before, the following new combinations are here proposed: *Quedius amamiensis* (Watanabe, 1990), comb. n.; *Quedius circumipectus* (Cho, 1996), comb. n.; *Quedius elongatus* (Naomi, 1986), comb. n.; *Quedius japonicus* (Watanabe, 1990), comb. n.; *Quedius pectinatus* (Sharp, 1874), comb. n.; *Quedius setosus* (Sharp, 1889), comb. n.; *Quedius simillimus* (Fairmaire, 1891), comb. n.To avoid the resulting homonyms, the name *Quedius japonicus* (Watanabe, 1990) (non *Quedius japonicus* Sharp, 1874) is replaced with the new name *Quedius watanabei* Solodovnikov, nom. n.,while the name *Quedius pectinatus* Lea, 1908 (non *Quedius pectinatus* (Sharp, 1874)) is replaced with the new name *Quedius arthuri* Solodovnikov, nom. n. New names are provided because neither of these two junior homonyms had available synonyms that could be valid names in new combinations.

## Species-level problems in *Heterothops* and *Quedius*

### On the synonymy of *Heterothops praevius* Erichson, 1839 and *Heterothops niger* Kraatz, 1868

Controversy over the status of *Heterothops praevius* and *Heterothops niger* had begun soon after the publication of the original description of *Heterothops niger*. Although already a few earlier authors considered *Heterothops niger* as a synonym of *Heterothops praevius* (e.g., Fauvel 1874; Fowler 1888; Ganglbauer 1895; Porta 1907), a predominant approach was to treat the former either as a distinct species, or as some kind of the intraspecific form of *Heterothops praevius*. A long history of this controversy is summarized in Israelson (1979) and Lott (2008). Israelson (1979), based on the detailed morphological examination of specimens from Sweden and survey of the literature covering other regions, came to the conclusion that *Heterothops praevius* and *Heterothops niger* differ slightly in the body coloration (*Heterothops praevius* is paler, while *Heterothops niger* is darker), distribution (*Heterothops praevius* has broader distribution, while *Heterothops niger* has narrower distribution within the range of *Heterothops praevius*) and ecology (*Heterothops praevius* is free living, while *Heterothops niger* is nidicolous). Lott (2008), based on the morphological examination of British material, also came to the conclusion that *Heterothops praevius* (paler) and *Heterothops niger* (darker) differ in coloration. However he denied the sharp ecological difference between these species defined as “free living *Heterothops praevius* *versus* nidicolous *Heterothops niger*”. Contrary to expectations, in his survey *Heterothops praevius* was found not only in free habitats but also in the badger setts, while *Heterothops niger* was found only in the mole nests. Israelson (1979) proposed to consider *Heterothops niger* as a subspecies of *Heterothops praevius*, that clearly was not a good decision for sympatric (and even syntopic) forms. Lott (2008) removed such inconsistency by stating that these sympatric taxa are two separate species, even though the morphological difference between them is very vague.

My examination of the abundant material identified by various people as both species from various parts of Denmark, and similar combined but sparser sample from various parts of Europe, reveals the following. Firstly, there is no such clear coloration difference (pale *versus* dark) as it was stated by Israelson (1979) or Lott (2008) for limited samples. Intermediately colored specimens that are hard to assign to either of these two (dark or pale) categories of coloration are not exceptional even among the Danish material alone. Secondly, consistently with Israelson (1979), there is no hiatus in a continuous variation of the structures of the aedeagus within the pool combining paler (presumable *Heterothops praevius*) and darker (presumable *Heterothops niger*) specimens. Therefore there are no genitalic characters that would break a combined sample of the putative *Heterothops praevius* and *Heterothops niger* into two or any other number of groups. Thus, no structural character supports the vague division between paler and darker specimens. With such a weak basis for morphological delineation of *Heterothops niger* from *Heterothops praevius*, secondary data like ecology or distribution become unreliable, while a synonymy of *Heterothops praevius* and *Heterothops niger* is considered a preferable solution that is followed in Assing and Schülke (2012).

#### Quedius meridiocarpathicus

Smetana, 1958

http://species-id.net/wiki/Quedius_meridiocarpathicus

##### Type material examined

*Lectotype* (here designated): ♂, **Slovakia:** “Slovakia mer. Kamen. Most 5.5.1955 Smetana 1955/ Quedius meridiocarpathicus s. Smetana det. 1957/ Lectotype Quedius meridiocarpathicus Smetana A. Solodovnikov des. 2009/ Quedius meridiocarpathicus Smetana A. Solodovnikov det. 2009” (cAS); *paralectotypes*: 3 ♂, 6 ♀, same data as in lectotype (2 ♂, 5 ♀ in cAS; 1 ♂, 1 ♀ in ZMUC); 1 ♂, “Slovakia mer. or. Slanec Smetana 1953/ Quedius meridiocarpathicus spec. n. det. A. Smetana/ Paralectotype Quedius meridiocarpathicus Smetana A. Solodovnikov des. 2009/ Quedius molochinus (Grav.) A. Solodovnikov det. 2009” (cAS).

##### Additional material examined

**Italy:** 1 ♂, Istria, Noghera (ZMUC); **Greece:** 1 ♂, 1 ♀, Parnass (ZMUC); 1 ♂, Janina, IV.1927, leg. C. Purkyně (ZMUC); **Turkey:** 1 ♂, Saray, 30 km W of Ankara, 23.II.1973 (ZMUC); **Bulgaria:** 1 ♂, Macedonia, Sandanski, 6–11.V.1984, leg. Wrase (cSch); 1 ♂, “Bulgaria, July 1975” (cSch); **Romania:** 1 ♂, 2 ♀, Eastern Romania, Mamaia/ Black Sea, 12–16.VII.1981, Wrase/Fietzke (cSch); **Ukraine:** 1 ♂, 1 ♀, Crimea, Simferopol, 30.III.1999 (cSch); 2 ♂, 2 ♀, Environs of Odessa, right bank of Kujalnitskij estuary, 10.VI.2005, under stones, leg. A. Gontarenko (ZMUC); **Russia:** Krasnodar territory: 2 ♂, 15 km S of vill. Taman’, 15.V.1995, sandy sea shore, under logs; 1 ♀, Karabetova Gryada 5 km SE of vill. Taman’, in litter at the bank of the permanent pond; 1 ♂, Mt. Tkhab, valley of river Zhene, 21.VI.1992, in forest litter, leg. M. Savitsky; 1 ♂, distr. of Tuapse, env. of vill. Massazhay, 15.III.1999, bank of river Tuapse, under stone, leg. K. Egorov; 1 ♀, distr. of Tuapse, env. of vill. Krasnoe, 17.III.1999, bottomland meadow of river Tuapse, under stone, leg. K. Egorov (ZMUC).

##### Discussion

**Figures 1–8.**
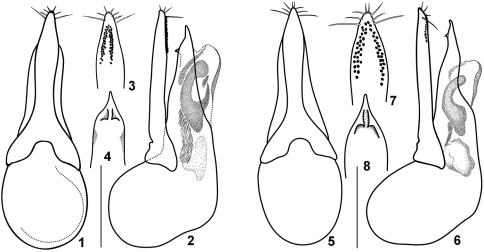
Details of the aedeagus of *Quedius molochinus* **1–4** and *Quedius meridiocarpathicus* **5–8**): **1, 5**, aedeagus dorsally (parameral side); **2, 6**, aedeagus laterally; **3, 7**, apical portion of paramere, side with sensory peg setae; **4, 8**, apical portion of median lobe, dorsal (parameral) side. Scale bars: 1 mm for **1, 2, 5, 6**; 0.8 mm for **3, 4, 7, 8**.

*Quedius meridiocarpathicus* Smetana, 1958 is very similar to *Quedius molochinus* (Gravenhorst, 1906). Both species can be reliably distinguished only by the shape of their aedeagi (Figs 1–8) and mostly by the shape of the largest (C-like) sclerite of the internal sac (cf. Figs 2 and 6). External characters hitherto used for separation of these species (details of punctuation of the elytra and abdomen, slight difference in the proportions of the body parts), as well as details of the shape of the aedeagus are variable in both species. Since some specimens of *Quedius meridiocarpathicus* in the collections are misidentified as *Quedius molochinus* and *vice versa*, the hitherto published distribution records for both of them (for a summary of literature see Herman 2001) in Central, Southern and Eastern Europe need revision. In fact, even the type series of *Quedius meridiocarpathicus* includes one male specimen of *Quedius molochinus*, an ambiguity here eliminated by designation of the lectotype (see below). Based on the material which I have examined (listed above, and more), *Quedius meridiocarpathicus* is reliably known from the south of Central and Eastern Europe, as well as from the Balkan Peninsula and Turkey.

##### Lectotype designation

The only information about the type material published in the original description of *Quedius meridiocarpathicus* is that it was collected at “Kamenný most” and “Slanec” in southern Slovakia (Smetana 1958). Aleš Smetana kindly sent me 11 specimens as a type series of *Quedius meridiocarpathicus*, all of them were collected by himself: 10 (4 males, 6 females) at Kamenný Most on 3.V.1955, and one male at Slanec in 1953. All these specimens are considered as syntypes. Of them, a single male from Slanec is undoubtedly *Quedius molochinus*, but all males from Kamenný Most belong to *Quedius meridiocarpathicus*. Females from Kamenný Most are also identified as *Quedius meridiocarpathicus* based on the association with the respective males. To avoid future ambiguity about the identity of *Quedius meridiocarpathicus* one male from Kamenný Most is here designated as a lectotype of this species.

#### Quedius pseudonigriceps

Reitter, 1909

http://species-id.net/wiki/Quedius_pseudonigriceps

Quedius noricus Bernhauer, 1927, syn. n.

##### Type material examined

*Quedius pseudonigriceps*: **Bosnia and Herzegovina:** *Lectotype* (here designated): ♂, “Nevesinje, V. Zoufal/ coll. Reitter/ Paratypus Quedius humeralis v. pseudonigriceps Reitter 1909” (HNHM); *paralectotypes*: 1 ♀, same data as in lectotype; 1 ♂, “Herzegovina Velež-Planina 1900 – 9/ Quedius humeralis Steph. coll. Reitter/ Q. (Sauridus) pseudonigriceps Reitt. H. Coiffait det. 1967” (HNHM); **Turkey:** 1 ♀, “Alem-Dagh/ coll. Reitter/ Holotypus Quedius humeralis var. pseudonigriceps Reitter 1909” (HNHM).

*Quedius noricus*: **Austria:** *Lectotype* (here designated): ♀, “Hofgastein tal Juli 1926 Bernhauer/ noricus Bernh. Typus [in Bernhauer’s handwriting]/ Chicago NH Mus M. Bernhauer Collection”; (FMNH); *paralectotype*: 1 ♀, “Hofgastein tal Juli 1926 Bernhauer/ noricus Bernh. Cotypus [in Bernhauer’s handwriting]/ Chicago NH Mus M. Bernhauer Collection” (FMNH).

##### Additional material examined

**Austria:** 1 ♂, “Gesteinertal Brugg AU 900 m Bernh/ Erlenlaub Juni 1928/ noricus Brnh. Det. Bernhauer [in Bernhauer’s handwriting]/ ex. Coll. Sceerpeltz” (NHMW); “ Gesteinertal Brugg AU 850 M. Bernh./ Erlenlaub 21.VI. 1928/ noricus Brnh. Det. Bernhauer [in Bernhauer’s handwriting]/ Chicago NH Mus M. Bernhauer Collection” (FMNH); 1 ♀, “Bad Brugg, Erlenlaub, VI.1936 Bernhauer/ Chicago NH Mus M. Bernhauer Collection” (FMNH); 1 ♀, “Badbruck, 900 m, VI.1930, Erienlaub/ Chicago NH Mus M. Bernhauer Collection” (FMNH); 1 ♀, “Gesteinertal Angertal VI.1929 Erle/ noricus Brnh. Det. Bernhauer [in Bernhauer’s handwriting]/ Dr. M. Bernhauer donavit/ ex. Coll. Sceerpeltz/ Cotypus Quedius noricus Bernhauer [pink label in Scheerpeltz’ handwriting]” (NHMW); 1 ♀, “Gesteinertal Angertal VI.1929 Erle / Chicago NH Mus M. Bernhauer Collection” (FMNH); 1 ♂, “Bad Gastein, Bad Bruck F. Leeder [Leder] leg./ Q. noricus det. F. Schubert” (NHMW); 1 ♂, “Gastein Umg. Saltsburg/ leg. Kaiser 6.1932/ Bruck/ noricus Bh. [not Bernhauer’s handwriting]” (NHMW); 1 ♀, “Hofgastein tal Juli 1926 Bernhauer/ noricus Bernh. Det. bernhauer [in Bernhauer’s handwriting]/ Chicago NH Mus M. Bernhauer Collection” (FMNH); 1 ♀, “Hofgastein Juli 1926 / noricus Bernh./ Chicago NH Mus M. Bernhauer Collection” (FMNH); **Bosnia and Herzegovina:** 2 ♂, 6 ♀, Majavica Bosna, VI. Zoufal (NMPC and ZMUC); 2 ♂, “Nevesinje, K. Kyselý”; **Republic of Macedonia:** 1 ♂, “AliBotuš VI.29 Maced. Mařan et Táborský lgt.” (NMPC); 1 ♂, “Maced. Perister Sv. Petka 7.14. Dr. Rambousek” (NMPC); 1 ♂, Maced. Galičica plan. VIII.1930, Dr. Rambousek (NMPC); **Romania:** 1 ♂, 7 ♀, Romania, Herculesbad (NMPC and ZMUC); 1 ♂, 2 ♀, Romania, Bălle Herculan, legt. Ing. Machulka (NMPC); **Hungary:** 1 ♂, Hungaria Com. Bihar, Dr. Fleischer (NMPC).

##### Discussion.

*Quedius noricus* was described from two females collected at “Hofgastein Tal Juli 1926 Bernhauer” [label data from two syntypes] in Austria (Bernhauer 1927). Coiffait (1963, 1978) included this species in his determination keys, provided its redescription and outlined its distribution as “Alpes orientales, montagnes d’Europe centrale” [Eastern Alps, mountains of Central Europe]. He provided illustrations of the aedeagus of this species based on the material from Chech Republic (“chaîne Bryb”). For some reason *Quedius noricus* was not included in the keys to the Central European Staphylinidae (Lohse 1964), but it was added there later (Lohse 1989), based on the mentioned accounts of this species by Coiffait (1963, 1978). Horion’s (1965) brief account about *Quedius noricus* was also based on the earlier published Bernhauer’s original description and data in Coiffait (1963) only. Additionally, based on the personal communications from Scheerpeltz and Korge, Horion (1965) mentioned some other specimens of *Quedius noricus* from Estern Alps (“Bad Gastein, Leder leg., det. Bernhauer (i.l.) Badbruck (900 m) [here examined, see above] und Kötschental (1300 m): Bernhauer leg.; Kolm-Saigurn Käufel leg.”: material from Scheerpeltz’ collection), and from the southern part of Romania (1 specimen from “Banat” identified by Korge based on the illustrations in Coiffait (1963). No other material identified as *Quedius noricus* was ever mentioned in the literature.

**Figures 9–11.**
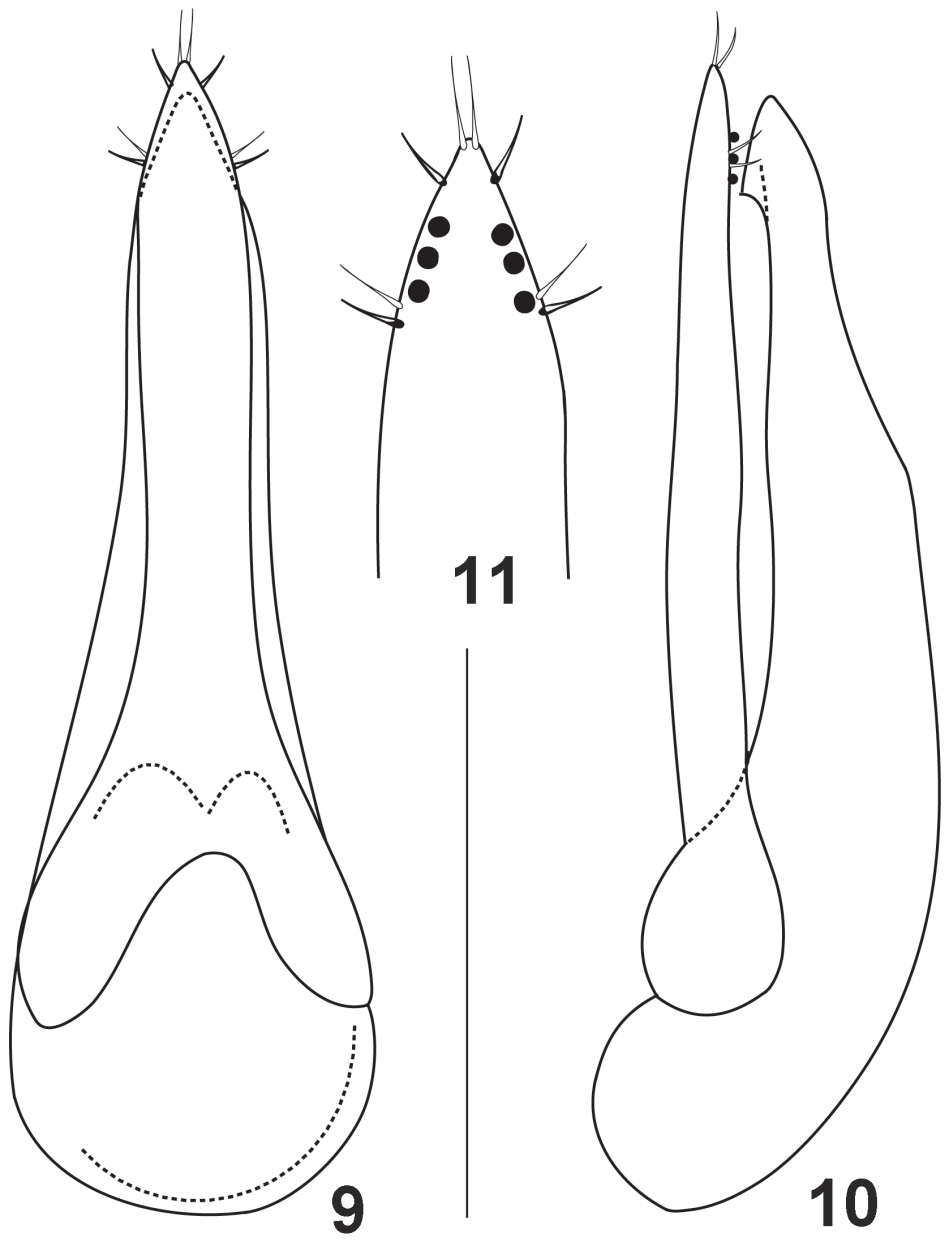
Aedeagus of *Quedius pseudonigriceps*: **9** dorsally (parameral side) **10** laterally **11** apical portion of paramere, side with sensory peg setae. Scale bar: 0.5 mm for **9, 10;** 0.25 mm for **11**.

It is difficult to establish the identity of two female syntypes of *Quedius noricus* because they belong to the complex of species (resembling *Quedius limbatus*) where the study of male genitalia is critical for the species identification. However, among the additional material from the Vienna Museum of Natural History (see above), there are three male specimens, one of which was identified by Bernhauer as *Quedius noricus*. Although neither of them are syntypes of *Quedius noricus*, they were collected near the type locality of that species. Examination of this valuable authentic material shows that *Quedius noricus* is conspecific with *Quedius pseudonigriceps* Reitter, 1909, the latter species earlier revised in Solodovnikov (2004). *Quedius pseudonigriceps* (Figs 9–11) is widely distributed in Southern Europe, Asia Minor, and Transcaucasia, while this new synonymy clarifies its distributionin the southern Central Europe.

##### Lectotype designation

To fix the identity of *Quedius noricus* Bernhauer, 1927, one of the syntypes (with the Bernhauer’s label “type”) is designated as a lectotype. The syntypes of *Quedius pseudonigriceps* were revised in Solodovnikov (2004), of them one male is designated here as a lectotype. Both lectotype designations are done for the unambiguous fixation of the names placed in synonymy.

#### Quedius maurorufus

(Gravenhorst, 1806)

http://species-id.net/wiki/Quedius_maurorufus

Quedius richteri Korge, 1966, syn. n.

##### Type material examined

*Quedius richteri*: Holotype: **Germany:** female, ”Stolpe a. Oder Uckermark, 1986/ Quedius (Sauridus) richteri Korge ♀ - Holotypus”; paratype: 1 male, “Stolpe/ Mark leg. D. Richter / Glykolfallen August 1965/ Paratypus Quedius richteri Korge” (cKrg). Additional specimen: 1 ?female [apex of the abdomen missing], same data as in paratype, marked as paratype [but not listed in the type series in the original description].

##### Remarks

**Figures 12–13.**
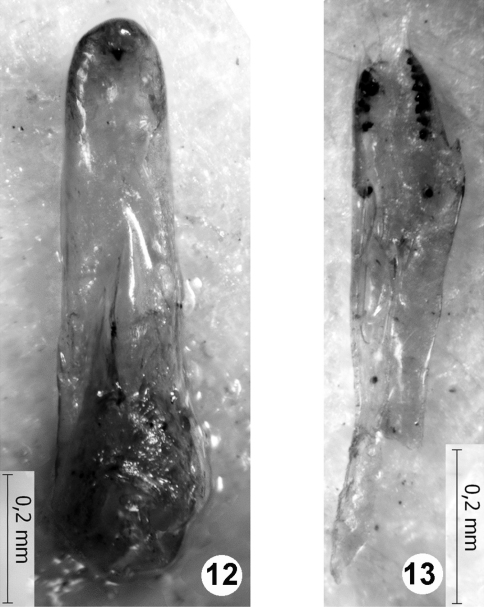
Aedeagus of the paratype of *Quedius richteri*: **12** median lobe dorsally (parameral side, paramere detached) **13** detached paramere, side with sensory peg setae. Scale bars: 0.2 mm.

The female holotype, the damaged male paratype (Figs 12, 13), and the unsexed specimen (without apex of abdomen, marked as “paratype” but not listed in the original description) are the only specimens known as *Quedius richteri* Korge, 1966. As stated in the original description of *Quedius richteri* (Korge 1966), and confirmed by the study of the type material here, externally this species is identical with *Quedius maurorufus* (Grav.). The only available male of *Quedius richteri* differs from *Quedius maurorufus* (Grav.) in the shape of the aedeagus (Figs 12, 13). The aedeagus of that single male of *Quedius richteri* shares the same structural plan with the aedeagus of *Quedius maurorufus,* and, at the same time, it displays some abnormal asymmetry. These facts, combined with the somewhat deformed external morphology of the corresponding male paratype of *Quedius richteri*, suggest that it is a teratological specimen of *Quedius maurorufus* (Grav.). Therefore, the name *Quedius richteri* Korge, 1966 is placed in synonymy with *Quedius maurorufus* (Gravenhorst, 1806), a wide-spread European species that is rather common in Central Europe. Lack of any other collecting events of *Quedius richteri*, described from the area of very strong entomological attention, is additional strong evidence for the mentioned teratology of *Quedius maurorufus* and resulting synonymy.

#### Quedius suturalis

Kiesenwetter, 1845

http://species-id.net/wiki/Quedius_suturalis

Quedius merlini Drugmand & Bruge 1991, syn. n.

##### Remarks.

*Quedius merlini* was described from three specimens (one male, two females) collected in Belgium (Tenneville, Fange Massa) in 1986 in a Lundgren trap (Drugmand and Bruge 1991). Unfortunately the type material of this species was not located at the Royal Institute of Natural Science at Brussels, but the original description and illustrations of *Quedius merlini* leave no doubts that those specimens are misidentified *Quedius suturalis* Ksw. After the description, *Quedius merlini* was never recorded again either in Belgium or anywhere else. For such an entomologically popular region as Central Europe, this is additional evidence that *Quedius merlini* is not a valid species.

## Supplementary Material

XML Treatment for Quedius meridiocarpathicus

XML Treatment for Quedius pseudonigriceps

XML Treatment for Quedius maurorufus

XML Treatment for Quedius suturalis

## References

[B1] Ashe JS Timm RM (1988) *Chilamblyopinus piceus*, a new genus and species of amblyopinine (Coleoptera: Staphylinidae) from southern Chile, with a discussion of amblyopinine generic relationships. Journal of the Kansas Entomological Society 61: 46-57.

[B2] Assing V Schülke M (2012) Freude–Harde–Lohse–Klausnitzer – Die Käfer Mitteleuropas. Band 4. Staphylinidae I. Zweite neubearbeitete Auflage. Heidelberg: Spektrum Akademischer Verlag.

[B3] Bernhauer M (1927) Neue Staphyliniden des paläarktischen Faunengebietes. Koleopterologische Rundschau 13 (2): 90-99.

[B4] Blackwelder RE (1952) The generic names of the beetle family Staphylinidae, with an essay on genotypy. Bulletin of the United States National Museum 200: 1-483.

[B5] Bouchard P Bousquet Y Davies AE Alonso-Zarazaga MA Lawrence JF Lyal CH Newton AF Reid CAM Schmitt M Ślipiński SA Smith ABT (2011) Family-group names in Coleoptera (Insecta). ZooKeys 88: 1-972. 10.3897/zookeys.88.807 PMC3088472 21594053

[B6] Chatzimanolis S Cohen I Schomann A Solodovnikov A. (2010) Molecular phylogeny of the mega-diverse rove beetle tribe Staphylinini (Insecta, Coleoptera, Staphylinidae). Zoologica Scripta 39 (5): 436-449. 10.1111/j.1463-6409.2010.00438.x

[B7] Coiffait H (1963) Les *Quedius* du sous-genre *Sauridus* de la région paléarctique occidentale (avec description de formes nouvelles) (Col. Staphylinidae). Bulletin de la Société d’Histoire Naturelle de Toulouse 98: 372-420.

[B8] Coiffait H (1978) Coléoptères Staphylinidae de la région paléarctique occidentale. III. Sous famille Staphylininae, tribu Quediini; sous famille Paederinae, tribu Pinophilini. Nouvelle Revue d’Entomologie, Supplement 8 (4): 1-364.

[B9] Drugmand D Bruge H (1991) A propos d’une nouvelle espèce de *Quedius* Stephens, 1832 découverte en Belgique (Coleoptera, Staphylinidae, Staphylininae). Bulletin et Annales de la Société Royale Belge d’Entomologie 127 (5/6): 191–197.

[B10] Erichson WF (1839) Die Käfer der Mark Brandenburg. Vol. 1, Part 2, F. H. Morin, Berlin, 385–740.

[B11] Fauvel A (1874) Faune Gallo-Rhénane ou description des insectes qui habitent la France, la Belgique, la Hollande, les provinces Rhénanes et le Valais, avec tableaux synoptiques et planches gravées (suite). Bulletin de la Société Linnéenne de Normandie 8(2): 167–318, pls. 1–2.

[B12] Fowler WW (1888) The Coleoptera of the British Islands. A descriptive account of the families, genera, and species indigenous to Great Britain and Ireland, with notes as to localities, habitats, etc. Vol. II. Staphylinidae. L. Reeve and Co., London, 444 pp., pls. 37–70.

[B13] Ganglbauer L (1895) Die Käfer von Mitteleuropa. Die Käfer der österreichisch-ungarischen Monarchie, Deutschlands, der Schweiz, sowie des französischen und italienischen Alpengebietes. Vol. 2, Familienreihe Staphylinoidea, I. Theil: Staphylinidae, Pselaphidae. Carl Gerold’s Sohn, Vienna, vi + 881 pp.

[B14] Herman L (2001) Catalog of the Staphylinidae (Insecta: Coleoptera). 1758 to the end of the second millennium. Parts I-VII. Bulletin of the American Museum of Natural History 265: 1-4218. [in 7 vols.]

[B15] Horion A (1965) Faunistik der mitteleuropäischen Käfer. Vol. 10: Staphylinidae, 2. Teil, Paederinae bis Staphylininae. A. Feyel, Überlingen-Bodensee, xv + 335 pp.

[B16] Israelson G (1979) On the taxonomy of some west European and Macaronesian *Heterothops* Stephens (Coleoptera: Staphylinidae). Entomologica Scandinavica 10 (4): 261-268. 10.1163/187631279X00141

[B17] Korge H (1966) [*Quedius (Sauridus) richteri*]. In: Korge H Schulze J (Eds). Beiträge zur Kenntnis der märkischen Koleopterenfauna (Teil XXIX). Mitteilungen der Deutschen Entomologischen Gesellschaft 25: 57–67.

[B18] Kraatz (1857) Naturgeschichte der Insekten Deutschlands, Abteilung 1, Coleoptera, vol. 2, Staphylinii. Lief. Nicolaische Buchhandlung, Berlin, 3–6, 377–1080.

[B19] Lacordaire JT (1854) Histoire naturelle des Insectes. Genera des Coléoptères ou exposé méthodique et critique de tous les genres proposés jusqu’ici dans cet ordre d’insects. Vol. 2, contenant les familles des Paussides, Staphyliniens, . .. Hétérocérides. Librairie Encyclopédique de Roret, Paris., 548 pp.

[B20] Leach WE (1819) [New genera]. In: Samouelle, G. The Entomologist’s Useful Compendium, or an Introduction to the Knowledge of British Insects, … Thomas Boys, London, 496 pp., 12 pls.

[B21] Lohse GA (1964) Band 4 Staphylinidae I (Micropeplinae bis Tachyporinae). In: Freude H Harde KW Lohse GA (Eds). Die Käfer Mitteleuropas. Goecke and Evers, Krefeld: 1-264.

[B22] Lohse GA (1989) Ergänzungen und Berichtigungen zu Band 4. 23. Familie Staphylinidae (I) (Piestinae bis Tachyporinae). In: Lohse GA Lucht WH (Eds). Die Käfer Mitteleuropas. 1. Supplementband mit Katalogteil, Goecke and Evers, Krefeld: 121-183.

[B23] Lott D (2008) Beetles from underground mammal nests in the East Midlands. Coleopterist 17: 101-114.

[B24] Newton AF Thayer MK (2005) Catalog of higher taxa of Staphyliniformia and genera and subgenera of Staphylinoidea. Field Museum of Natural History, Chicago [online]. http:// www.fieldmuseum.org/peet_staph/db_1a.html [last accessed on November 10, 2011]

[B25] Paulian R (1941) Les premiers états des Staphylinoidea (Coleoptera). Étude de morphologie comparée. Mémoires du Muséum National d’Histoire Naturelle (NS) 15: 1–361, pls. 1–3.

[B26] Pietrykowska-Tudruj J Staniec B Solodovnikov AY (2011) Discovery of the *Quedius antipodum* sharp larva from New Zealand: phylogenetic test of larval morphology for Staphylinini at the intratribal level (Coleoptera: Staphylinidae). Systematic Entomology. 10.1111/j.1365-3113.2011.00612.x

[B27] Porta (1907) Revisione degli stafilinidi italiani. IIIa parte. Quediini. Rivista Coleotterologica Italiana 5(4): 85–116, (5): 125–153.

[B28] Pototskaya VA (1967) Classification key of the larvae of Staphylinidae in the European part of the USSR. Nauka, Moscow, 120 pp.[in Russian]

[B29] Schaum HR (1859) Catalogus Coleopterorum Europae. In: Verbindung mit Dr. G. Kraatz und H. v. Kiesenwetter. Nicolaische Verlagsbuchhandlung, Berlin, iv + 121 pp.

[B30] Sharp D (1884) Fam. Staphylinidae, pp. 313–392, pls. 8–9. In: Biologia Centrali-Americana. Insecta, Coleoptera. Vol. 1(2). Taylor and Francis, London, xv + 824 pp., 19 pls.

[B31] Smetana A (1958) Drabčíkovití - Staphylinidae I, Staphylininae (Řád: Brouci - Coleoptera). In: Fauna ČSR. Vol. 12. Ceskoslovenské Akademie Ved, Praha, 435 pp.

[B32] Smetana A (1971) Revision of the tribe Quediini of America north of Mexico (Coleoptera: Staphylinidae). Memoirs of the Entomological Society of Canada 79: vi + 303 pp.

[B33] Smetana A (1988) Revision of the tribes Quediini and Atanygnathini. Part II. The Himalayan region (Coleoptera: Staphylinidae). Quaestiones Entomologicae 24: 163-464.

[B34] Smetana A (1995) Revision of the tribes Quediini and Tanygnathinini. Part III. Taiwan (Coleoptera: Staphylinidae). Special Publication Number 6, National Museum of Natural Science, Taichung, Taiwan, ROC, 145 pp.

[B35] Smetana A (2004): Staphylinidae except Pselaphinae and Scaphidiinae, P. 237–698 In: Löbl, Smetana A (Eds). Catalogue of Palaearctic Coleoptera. Volume 2. Hydrophiloidea-Histeroidea-Staphylinoidea. Stenstrup: Apollo Books: 1-942.

[B36] Smetana A Davies A (2000) Reclassification of the north temperate taxa associated with *Staphylinus* sensu lato, including comments on relevant subtribes of Staphylinini (Coleoptera: Staphylinidae). American Museum Novitates 3287: 1-88. 10.1206/0003-0082(2000)287<0001:ROTNTT>2.0.CO;2

[B37] Solodovnikov AY (2004) Taxonomy and faunistics of some West Palaearctic *Quedius* Stephens subgenus Raphirus Stephens (Coleoptera: Staphylinidae). Koleopterologische Rundschau 74: 221-243.

[B38] Solodovnikov AY (2005) *Natalignathus*, gen. n. and larvae of *Atanygnathus*: a missing phylogenetic link between subtribes Quediina and Tanygnathinina (Coleoptera: Staphylinidae: Staphylininae: Staphylinini). Invertebrate Systematics 19: 75-98. 10.1071/IS04031

[B39] Solodovnikov AY (2006) Revision and phylogenetic assessment of *Afroquedius* gen. n. from South Africa: toward new concepts of the genus *Quedius*, subtribe Quediina and reclassification of the tribe Staphylinini (Coleoptera: Staphylinidae: Staphylininae). Annals of the Entomological Society of America 99 (6): 1064-1084. 10.1603/0013-8746(2006)99[1064:RAPAOA]2.0.CO;2

[B40] Solodovnikov AY Newton AF (2005) Phylogenetic placement of Arrowinini trib. n. within the subfamily Staphylininae (Coleoptera: Staphylinidae), with revision of the relict South African genus *Arrowinus* and description of its larva. Systematic Entomology 30: 398-441. 10.1111/j.1365-3113.2004.00283.x

[B41] Solodovnikov AY Newton AF (2009) *Australotarsius* - a new genus of the rove beetle tribe Staphylinini from Australia (Coleoptera: Staphylinidae: Staphylininae). Zootaxa 2033: 49-57. 10.1111/j.1365-3113.2008.00468.x

[B42] Solodovnikov A Schomann A (2009) Revised systematics and biogeography of “Quediina” of Subsaharan Africa: new phylogenetic insights into the rove beetle tribe Staphylinini (Coleoptera: Staphylinidae). Systematic Entomology 34: 443-446.

[B43] Staniec B (2005) Description of the developmental stages of *Atanygnathus terminalis* (Erichson, 1839) (Coleoptera, Staphylinidae, Staphylininae), with comments on its biology. Deutsche Entomologische Zeitschrift 52: 173 – 190. 10.1002/mmnd.200410011

[B44] Strassen R zur. (1957) Zur Oekologie des *Velleius dilatatus* Fabricius, eines als Raumgast bei *Vespa crabro* Linnaeus lebenden Staphyliniden (Ins. Col.). Zeitschrift für Morphologie und Ökologie der Tiere 46 (3): 243-292. 10.1007/BF00383800

[B45] Westwood JO (1838) Synopsis of the genera of British insects. An introduction to the modern classification of insects, founded on the natural habits and corresponding organization of the different families. Longman, Orme, Brown, Green and Longmans, London, 1–48.

